# Nisin A Treatment to Protect Honey Bee Larvae from European Foulbrood Disease

**DOI:** 10.1007/s12602-025-10450-4

**Published:** 2025-01-15

**Authors:** Keiko Nakamura, Daisuke Takamatsu, Mariko Harada, Takeshi Zendo, Yuka Sekiya, Akihito Endo

**Affiliations:** 1 Research and Business Promotion Division, Research Institute for Animal Science in Biochemistry and Toxicology, Sagamihara, Kanagawa 252-0132 Japan; 2 Division of Infectious Animal Disease Research, National Institute of Animal Health, National Agriculture and Food Research Organization, Tsukuba, Ibaraki 305-0856 Japan; 3 Division of Systems Bioengineering, Department of Bioscience and Biotechnology, Faculty of Agriculture, Graduate School, Kyushu University, Fukuoka, Fukuoka 819-0395 Japan; 4 Tokyo Metropolitan Livestock Hygiene Service Center, Hinode, Tokyo 190-0182 Japan; 5 Department of Nutritional Science and Food Safety, Faculty of Applied Bioscience, Tokyo University of Agriculture, Setagaya, Tokyo 156-8502 Japan

**Keywords:** Honey bee larvae, *M. plutonius*, Nisin A, Antagonistic activity, Toxicity

## Abstract

European foulbrood (EFB) is a bee larvae-specific infectious disease and the causative pathogen is *Melissococcus plutonius*. Broad-spectrum antibiotics have classically been used in many countries to control the pathogens; however, their use in apiaries was discontinued in several countries due to concerns regarding the health of bees and humans. Therefore, the development of alternative treatments for use in apiaries that are safe for bees and humans is essential. The present study examined the effects of nisin A supplementation using artificially reared honey bee larvae infected by *M. plutonius* strains. The results obtained showed that a non-purified nisin A product was toxic to honey bee larvae, while semi-purified nisin A by removing low-molecular-weight (< 3,000) chemicals was not lethal to honey bee larvae. A larval diet supplemented with the semi-purified nisin A significantly increased the survival rate of larvae infected by *M. plutonius*. The levels of nisin A required for this rescue differed between the *M. plutonius* strains used for infection, and 12.5 and 100 µg/mL were required for strain DAT606 belonging to clonal complex 3 and strain DAT561 belonging to clonal complex 12, respectively. This beneficial effect was attributed to the antagonistic activity of nisin A against *M. plutonius* strains, and the levels of viable *M. plutonius* strains significantly decreased in the larval gut at the required concentrations. Due to the risks associated with the use of antibiotics in apiaries, food-grade nisin A is a promising alternative to control EFB.

**Supplementary Information:**

The online version contains supplementary material available at 10.1007/s12602-025-10450-4.

## Introduction

Western honey bees (*Apis mellifera* L.) are one of the most important pollinators in nature and crop production. They greatly contribute to the production of food consumed by humans, with approximately 35% of the human diet estimated to come from the direct or indirect outcomes of honey bee pollination [1]. However, these crucial agricultural insects face several threats in nature, including climate change, pathogens, parasites, and pesticides [2].

European foulbrood (EFB) is a bee larvae-specific infectious disease, and EFB outbreaks have been reported in many countries worldwide [3, 4]. *Melissococcus plutonius* is a causative pathogen of EFB. It is a honey bee-specific microbe that has been detected in honey bee habitats, such as honey and hive debris [5, 6]. Strains of this pathogen have been divided into several genomic groups, called clonal complex (CC), based on multi-locus sequencing typing profiling, and their pathogenicity differs among CCs [3, 4, 7, 8]. Known *M. plutonius* strains are grouped into CC3, CC12, or CC13. Strains in CC12 are extremely virulent and kill > 70% of infected larvae within 5–6 days in many in vitro exposure bioassays [4, 8]. Strains in CC3 are less virulent (≈60% larvae die within 5–6 days), and infections caused by CC13 strains have been reported to only slightly affect the survivability of larvae. Recently examined strains in Japan, Mexico, and Canada were mainly grouped into CC12, while those found in the UK and Switzerland mainly belonged to CC3 and CC13 [3, 4, 9]. Previous studies have reported that > 85% of honey samples tested were contaminated with *M. plutonius* in European countries and Japan [5, 10]. In addition, the pathogen has been detected in adult bees and larvae in colonies without visual EFB symptoms [11–13], suggesting that the risk of EFB may be permanent in all apiaries. To control pathogens, broad-spectrum antibiotics that mainly target *M. plutonius* and the American foulbrood (AFB) pathogen *Paenibacillus larvae* have classically been used in many countries. Oxytetracycline is only the antibiotic approved for EFB in the USA, while oxytetracycline, tylosin, and lincomycin are used to control AFB [14]. In Japan, two macrolide antibiotics, mirosamicin and tylosin, have been used as prophylactics for AFB [15]. However, the application of these antibiotics causes detrimental effects on the health of both bees and humans. These include the accumulation of antibiotic resistance genes in the honey bee gut microbiota [16], the generation of antibiotic-resistant pathogens [15, 17], increased honey bee mortality rates [18], and the accumulation of antibiotic residues in honey consumed in the human diet [19, 20]. Therefore, the use of antibiotics in apiaries has been discontinued in several countries, particularly in the European Union [21]. Given these concerns, the development of alternative treatments for use in apiaries that are safe for bees and humans is essential for the production of safe diets and for the promotion of animal welfare.

Nisin A is a ribosomally synthesized antimicrobial peptide, i.e., bacteriocin, which is produced by specific strains of *Lactococcus lactis* and exhibits antimicrobial activity against various Gram-positive food-spoilage bacteria and animal pathogens [22–24]. It contains post-translationally modified unusual amino acids and is grouped into lantibiotics (lanthipeptides). Nisin A exhibits antimicrobial activity through the inhibition of cell wall biosynthesis by scavenging of the peptidoglycan precursor lipid II as well as lysis of the cell membrane by the formation of pores [25]. Nisin A has been globally used as a food preservative because of its potent bactericidal activity, in vivo safety tests, potential consumption history, and inactivation by human digestive enzymes [26, 27], whereas applicable foods and the approved levels of nisin A vary by country [26]. Moreover, nisin A and its variants are regarded as safe alternatives to antibiotics for clinical purposes [28]; however, its potential application to the control of honey bee pathogens has yet to be studied.

Therefore, the present study examined the effects of nisin A supplementation on *M. plutonius* infected honey bee larvae reared under artificial conditions. Larvae were infected by *M. plutonius* strains belonging to different CCs in vitro and their diets were supplemented with nisin A. The survivability of larvae was monitored over three weeks.

## Materials and Methods

### Bacterial Strains and Culture Conditions

The bacterial strains used in the present study are listed in Table 1. *Melissococcus plutonius* strains were isolated from EFB-diseased larvae of *A. mellifera* and *Apis cerana japonica* in Japan [7, 29]. These strains were cultured on KSBHI agar at 35 °C for five days under anaerobic conditions. The composition of KSBHI agar was described elsewhere [7]. *Clostridium perfringens* CP1 and *Escherichia coli* ATCC 25922 were cultured on modified GAM agar (Nissui, Japan) under anaerobic conditions and BHI agar (Becton Dickinson, Sparks, MD) under aerobic conditions, respectively, at 37 °C for 24 h. *Lapidilactobacillus dextrinicus* JCM 5887^ T^ was cultured in MRS broth (Beckton Dickinson) at 30 °C for 24 h. They were used as controls for nisin activity.

**Table 1:** Antimicrobial activity of nisin A

Indicator strains	MCI (μg/mL)
*Melissococcus plutonius* DAT606 (CC3) *Melissococcus plutonius* DAT968 (CC3) *Melissococcus plutonius* DAT561 (CC12) *Melissococcus plutonius* DAT892 (CC12) *Melissococcus plutonius* DAT985 (CC12) *Melissococcus plutonius* DAT580 (CC13) *Melissococcus plutonius* DAT869 (CC13) *Clostridium perfringens* CP1 *Escherichia coli* ATCC 25922	3.125 3.125 3.125 3.125 6.25 3.125 6.25 0.39 ND*

^*^ND, not determined. The strain exhibited resistance to the maximum concentration (200 µg/mL) used in the test

### Preparation of Nisin A Solution and Determination of Minimum Concentrations for Inhibition

The Nisin product was purchased from Merck Millipore (Germany) and contained 2.5% (w/w) nisin A with balance sodium chloride and denatured milk solids (https://pubchem.ncbi.nlm.nih.gov/substance/24897751). It was dissolved in deionized water at a concentration of 10 mg/mL containing 250 µg/mL nisin A, which was referred to as a non-purified nisin A solution. Five milliliters of the non-purified nisin A solution was then partially purified with a centrifugal ultrafiltration device (Amicon Ultra-15 3,000 NMWL, Merck Millipore), and 500 µL of semi-purified nisin A (containing 2.5 mg/mL nisin A) solution was obtained as the retentate. To confirm the recovery ratio of nisin A, the antagonistic activities of the semi-purified nisin A solution and flow-through were tested using the spot-on-lawn method with the nisin A-sensitive organism, *L. dextrinicus* JCM 5887^ T^, as previously described [23].

The semi-purified nisin A solution was used to assess the minimum concentrations for inhibition (MCI) of *M. plutonius* strains and to assess exposure bioassays using in vitro reared *A. mellifera* larvae. Since some *M. plutonius* strains were previously shown to grow poorly in liquid broth [3], the broth microdilution method to determine minimum inhibitory concentrations of antimicrobials was not appropriate for evaluating antagonistic activity against the strains tested. Therefore, to determine MCI, the semi-purified nisin A solution was subjected to a twofold serial dilution with sterile water and then used in the spot-on-lawn antimicrobial assay [30]. Briefly, *M. plutonius* strains were pre-cultured on KSBHI agar, as described above. Cells were recovered from pre-cultured agar and suspended in sterile water at O.D._600 nm_ = 1. The suspended cells (100 µL) were inoculated onto KSBHI agar (approx. 20 mL), and 10 µL of the serially diluted semi-purified nisin A solution was spotted onto the inoculated agar. The spotted agar media were cultured at 35 °C for 5 days under anaerobic conditions to observe a clear zone of inhibition. *Clostridium perfringens* CP1 strain and *E. coli* ATCC 25922 strain were included in the MCI test as a sensitive strain and resistant strain, respectively. These strains were cultured and the colonies that developed were used as inocula, as described above, except that the bacterial suspensions for inocula were adjusted to a concentration of O.D._600 nm_ = 0.2. The suspended cells (100 µL) were inoculated onto their appropriate agar media (approx. 20 mL), and 10 µL of the serially diluted semi-purified nisin A solution was spotted onto the inoculated agar.

### Exposure Bioassays Using In Vitro Reared *A. mellifera* Larvae

The nisin product used in the present study contained large amounts (> 97% w/w) of sodium chloride and denatured milk solids with nisin A. Sodium chloride may adversely affect the development of honey bee larvae, which normally eat food with a high potassium concentration [31–33]. Therefore, the effects of the non-purified nisin A product on bee larvae were initially investigated without *M. plutonius* infection in bioassay I (conducted in August 2022), and those of the semi-purified nisin A were tested in larvae infected by *M. plutonius* in bioassay II (conducted in in November 2023 and April 2024), independently. The strains* Melissococcus plutonius* DAT561 (belonging to CC12) and DAT606 (belonging to CC3) were used in bioassay II.

Larvae younger than 24-h-old were taken from clinically healthy colonies maintained in the Research Institute for Animal Science in Biochemistry and Toxicology, Sagamihara, Japan, and were grafted onto royal jelly in sterile Petri dishes using a grafting tool. Each larva in the dishes was transferred and reared in a grafting cell placed in individual wells of a 48-well cell culture plate. In each assay, larvae were collected from three different queens and randomly divided into test groups. Culture plates were kept at 34.5 ± 0.5 °C with a relative humidity of 95 ± 5% in an incubator until day 6 post-grafting (PG) [34]. Larvae were fed an artificial diet once a day until day 5 PG, except for day 1 PG (Supplementary Table S2). On and after day 7 PG, culture plates were further incubated in a desiccator at 34.5 ± 0.5 °C with a relative humidity of 80%. On day 14 PG, each plate was transferred to an emergence box in a desiccator and incubated at 34.5 ± 0.5 °C with a relative humidity of 50–80% until day 21 PG.

To prepare inocula, *M. plutonius* colonies on KSBHI agar were suspended in sterile water at a final concentration of approximately 0.8–3.2 × 10^7^ colony forming units (CFU)/mL, and the suspension was then mixed with artificial diet B' (Supplementary Table S3) at a ratio of 1:9 for inocula containing *M. plutonius* at 0.8–3.2 × 10^6^ CFU/mL. The final bacterial concentration in each inoculum was measured by plating serial dilutions of inocula onto KSBHI agar plates and counting colonies on the plates after an incubation at 34.5 °C for 5 days or longer under anaerobic conditions. Larvae in infected groups in bioassay II were fed 20 µL of the inocula on day 2 PG. In some groups, a solution of non-purified nisin A product or semi-purified nisin A was added to diet C and fed to larvae from days 3 to 5 PG (Supplementary Tables S2 and S3). Daily rations and formulas of the artificial diet are shown in Supplementary Tables S2 and S3, respectively.

In both bioassays I and II, mortalities were confirmed and recorded at the time of feeding from day 3 PG to day 7 PG and on day 14 PG, and dead larvae were systematically removed from the plates for sanitary reasons. A number of hatched adult bees and dead ones were recorded on day 21 PG. In the bioassay II, larval weights were measured in five live larvae per group on day 5 PG. Moreover, the viable cell number of *M. plutonius* strains in larvae was also studied in the five live larvae per group on day 5 PG using culturing techniques, as described previously [35].

### Statistical Analysis

Differences in the survival of tested larvae were analyzed by the Log-rank test with multiple comparisons (Bonferroni correction). Larval weights and logarithmic transformed bacterial loads in infected larvae were analyzed by a one-way ANOVA with Tukey’s multiple comparison test. All tests were performed using R version 3. 5. 3. (The R Foundation for Statistical Computing, Vienna, Austria) and EZR (Saitama Medical Center, Jichi Medical University, Saitama, Japan) [36], which is a graphical user interface for R. A value of *P* < 0.05 was considered as the threshold for significance.

## Results

### Effects of the Non-purified Nisin A Product on the Survivability of Non-infected Larvae

**Fig. 1:**
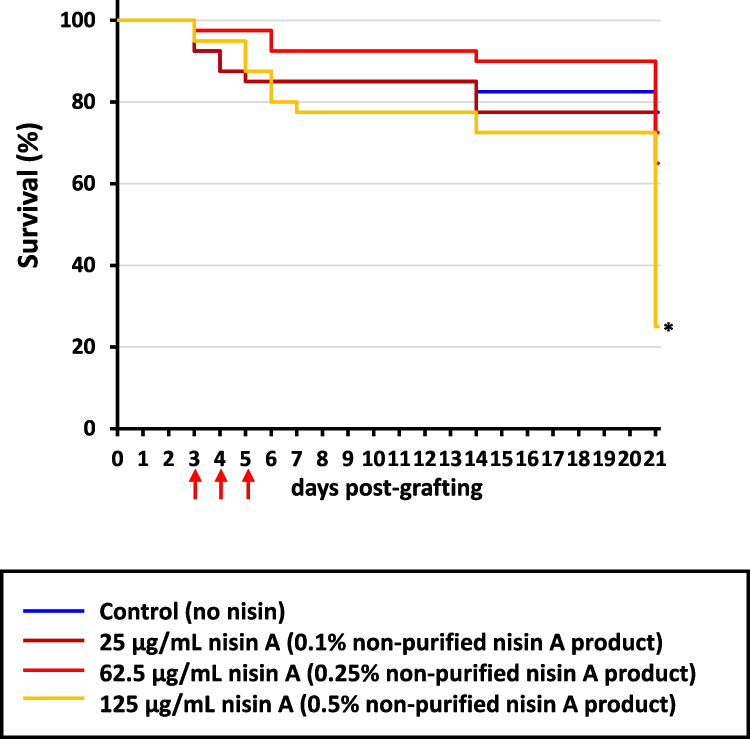
Survival rates of honey bee larvae administered different amounts of the non-purified nisin A product without infection. Red arrows indicate days for the nisin A treatment. Differences in the survival rate of larvae were statistically analyzed by the Log-rank test with multiple comparisons by the Bonferroni correction. The asterisk indicates a group in which the survival rate was significantly different (*P* < 0.001) from that of the control

The effects of the non-purified nisin A product on bee larvae were initially investigated by the addition of different concentrations to the diet of non-infected larvae (Exposure bioassay I in Supplementary Table S1). The survival rate of honey bee larvae was 77.5% on day 21 PG, when nisin A was not included in the diet (Fig. 1). In comparison with the control, the larval survival rate was not significantly different following supplementation with 0.1% and 0.25% non-purified nisin A product (nisin A concentrations of 25 and 62.5 µg/mL, respectively) (*P* > 0.05), with survival rates of 65 and 72.5%, respectively, on day 21 PG. On the other hand, survival rates were significantly lower following supplementation with 0.5% non-purified nisin A product (nisin A concentration of 125 µg/mL) than under other conditions (*P* < 0.001), reaching 25% on day 21 PG, suggesting that the non-purified nisin A product above a certain concentration was toxic to larvae.

### MCI of Semi-purified Nisin A Solution

The flow-through fraction obtained from the semi-purification of the nisin A product showed minor antagonistic activity against the indicator strain of *L. dextrinicus*, whereas the semi-purified fraction, concentrated tenfold, exhibited strong antagonistic activity against this strain. These results suggest that the majority of nisin A was recovered in the semi-purified nisin A solution, which contained 2.5 mg/mL nisin A.

The semi-purified nisin A exhibited potent antimicrobial activity against the sensitive strain of *C. perfringens* CP1, with MCI of 0.39 µg/mL nisin A (Table 1). The nisin-resistant strain of *E. coli* ATCC 25922 showed resistance to ≥ 200 µg/mL nisin A. *M. plutonius* strains belonging to different CCs exhibited similar sensitivity to the semi-purified nisin A. They were sensitive to ≥ 3.125 µg/mL nisin A, while strains DAT985 and DAT869 were sensitive to ≥ 6.25 µg/mL nisin A (Table 1).

### Prophylactic Application of Semi-purified Nisin A Solution for Larvae Infected by *M. plutonius* Strains

**Fig. 2:**
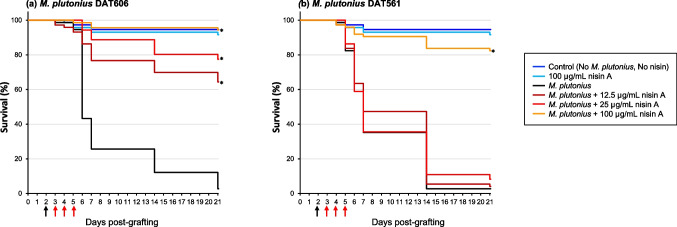
Survival rate of honey bee larvae infected by *M. plutonius* DAT606 (**a**) and DAT561 (**b**) and administered different amounts of semi-purified nisin A. Black arrows indicate days for *M. plutonius* infection, and red arrows indicate days for the nisin A treatment. Differences in the survival rate of larvae were statistically analyzed by the Log-rank test with multiple comparisons by the Bonferroni correction. Asterisks indicate infection groups treated with nisin A in which the survival rate was significantly higher (*P* < 0.001) than that in the group infected with *M. plutonius* and not treated with nisin A

Since the in vitro exposure bioassay II study was independently repeated twice as described in “Materials and Methods,” the results obtained were combined to normalize data. The semi-purified nisin A solution was supplied at different concentrations to larvae with or without infection by *M. plutonius* (Exposure bioassay II in Supplementary Table S1). Without infection, 98 and 93% of larvae survived supplementation with no and 100 µg/mL nisin A, respectively, on day 21 PG, with no significant differences (*P* = 1.00) (Fig. 2).

Following infection by *M. plutonius* DAT606 (CC3), the survival rate by the day 21 PG was 3%. The survival rate significantly increased to 65, 77, and 94% following supplementation with 12.5, 25, and 100 µg/mL nisin A (*P* < 0.001), respectively (Fig. 2a). Infection by *M. plutonius* DAT561 (CC12) significantly reduced the survival rate of larvae, which was only 3% on day 21 PG (Fig. 2b). Supplementation with 12.5 and 25 µg/mL nisin A did not significantly increase the survival rate, whereas the application of 100 µg/mL nisin A resulted in a significantly increased survival rate to 82% (*p* < 0.001). No statistical differences were found in survival rates between the control groups (i.e., no-infection-larvae groups with or without 100 µg/mL nisin A) and the DAT561 infection with 100 µg/mL nisin A-treated group (*P* > 0.2) (Fig. 2b).

**Fig. 3:**
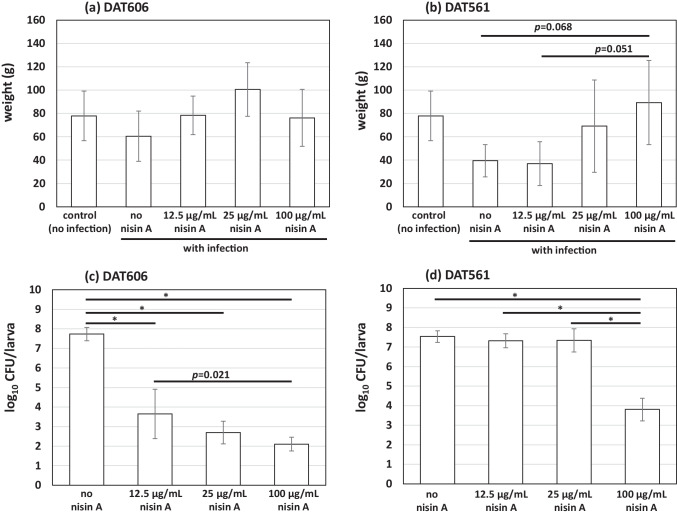
Weight of larvae (**a**, **b**) and CFU of *M. plutonius* in larvae (**c**, **d**) (*n* = 5) infected by DAT606 (**a**, **c**) and DAT561 (**b**, **d**). Differences in weight and CFU were statistically analyzed by a one-way ANOVA with Tukey's multiple comparison test. *P < 0.001

In terms of larval weights, no significant differences were found in the DAT606 infection groups, regardless of infection or the nisin A treatment on day 5 PG (Fig. 3a). In the DAT561 infection groups, larval weights were slightly heavier tendencies in the 100 µg/mL nisin A-treated group than in the no or 12.5 µg/mL nisin A-treated group (*P* < 0.07, Fig. 3b). Viable cells of *M. plutonius* were not recovered from the non-infected control group on day 5 PG. In the DAT606 infection groups, the viable cell number (log_10_CFU/larva) was 7.73 ± 0.34 (average ± standard deviation, SD) in the no nisin treatment group (Fig. 3c). This number significantly decreased to 3.65 ± 1.26, 2.69 ± 0.58, and 2.10 ± 0.36 in groups treated with 12.5, 25, and 100 µg/mL nisin A (*P* < 0.001), respectively (Fig. 3c). The treatment with 100 µg/mL nisin A reduced the CFU of DAT606 significantly more than the treatment with 12.5 µg/mL nisin A (*P* = 0.021). In the DAT561 infection groups, the viable cell number (log_10_CFU/larva) was 7.53 ± 0.30 (average ± SD) in the no treatment group (Fig. 3d). The cell number was not markedly affected by supplementation with 12.5 or 25 µg/mL nisin A, i.e., 7.32 ± 0.36 and 7.34 ± 0.34, respectively (*P* = 1.0). However, it was significantly decreased to 3.80 ± 0.58 by supplementation with 100 µg/mL nisin A (*P* < 0.001) (Fig. 3d).

## Discussion

In addition to its use as a food preservative, nisin has been incorporated into several products to control undesirable microbes. Nisin-containing dairy wipes are available for veterinary purposes to control bovine mastitis in the US [37] since several Gram-positive bacteria belonging to the families *Enterococcaceae, Staphylococcaceae,* and *Streptococcaceae* have been identified as causative pathogens. Toothpaste containing nisin A is available to control Gram-positive periodontal disease- and caries-related bacteria (https://oralpeace.com/en) [38]. Moreover, a recent study described a nisin-based plant therapy to suppress *Xylella fastidiosa* infection *in planta* [39]. The EFB causative pathogen *M. plutonius* is a Gram-positive microbe and is phylogenetically related to *L. lactis*; therefore, it is a potential target of nisin A.

The administration of 0.5% non-purified nisin A product (nisin A concentration of 125 µg/mL) significantly reduced the survival rate of honey bee larvae. The nisin A product used (Merck Millipore) contained high levels of sodium chloride based on the product information. High levels of sodium chloride may have decreased the survival rate of larvae. After the removal of low-molecular-weight (< 3000) chemicals, the supplementation of larval food with nisin A at a concentration of 100 µg/mL did not affect larval survivability, suggesting that 100 µg/mL nisin A is not a lethal stress to honey bee larvae.

The pathogenicity of *M. plutonius* strains in larvae was previously shown to be potent [8, 35]. The survival rates of larvae infected by *M. plutonius* DAT606 (CC3) and DAT561 (CC12) were very low, while the survival rates significantly improved by the administration of nisin A with different levels. The viable cell numbers of *M. plutonius* were consistent with these results. The CFU of *M. plutonius* in the DAT606 infection group were significantly lower with the 12.5, 25, and 100 µg/mL nisin A treatments than without the nisin A treatment. In the DAT561 (CC12) infection group, the 12.5 and 25 µg/mL nisin A treatments did not affect the CFU of DAT561 (CC12), while the 100 µg/mL nisin A treatment significantly reduced the CFU. These results suggest that improvements in survivability by supplementation with nisin A were due to the antimicrobial activity of nisin A against *M. plutonius* strains in larvae. The different levels of semi-purified nisin A required to decrease CFU between *M. plutonius* strains were not explained by the nisin A sensitivity of these strains, since they had the same MCI of 3.125 µg/mL nisin A.

In in vitro exposure bioassays, the level of nisin A required to control DAT561, i.e., 100 µg/mL, was markedly higher than the MCI of the strain (3.125 µg/mL). This result indicates that the larval diet or larval gut decreases the antagonistic activity of nisin A. One reason may be the degradable property of nisin A by digestive enzymes in the gastrointestinal tracts [40]. This degradable property is both a merit because it decreases the opportunity for resistant strains to emerge and a demerit due to its instability in nature. Since several adult honey bee commensals are sensitive to nisin A [23], the effects of nisin A treatments on the gut microbiota in honey bees have to be studied in future studies. A former study reported that exposure to flumethrin in honey bees significantly reduced α-diversity of gut microbiota, which resulted in changes of metabolic properties in the gut microbiota [41]. Moreover, since the administration of antibiotics to larvae is generally via honey or royal jelly fed to larvae by nurse bees, it is necessary to confirm whether nisin A can be stably delivered to larvae via these routes. The nisin A used in the present study was a chemical-grade product, which would be costly to apply in apiaries. One of alternative ways would be an application of nisin-producing *L. lactis* strains. Further studies are essential for assessing the effects of nisin A on the health of adults and larvae in hives.

## Conclusion

The reagent-grade Nisin A product was toxic to honey bee larvae, possibly due to the high concentration of salt present in the product. After the removal of low-molecular-weight (< 3000) chemicals, the semi-purified nisin A did not exhibit toxicity against honey bee larvae. The inclusion of semi-purified nisin A increased the survival rate of honey bee larvae infected by *M. plutonius* strains, while the nisin A concentration required differed between *M. plutonius* strains. Due to the present risks associated with the use of antibiotics in apiaries, food-grade nisin A is a promising alternative for the control of EFB.

## Supplementary Information

Below is the link to the electronic supplementary material.

Supplementary file1 (XLSX 15 KB)

## Data Availability

No datasets were generated or analysed during the current study.
